# Comparison of the in-vivo effect of two tranexamic acid doses on fibrinolysis parameters in adults undergoing valvular cardiac surgery with cardiopulmonary bypass - a pilot investigation

**DOI:** 10.1186/s12871-021-01234-8

**Published:** 2021-02-02

**Authors:** Zhen-feng Zhou, Wen Zhai, Li-na Yu, Kai Sun, Li-hong Sun, Xiu-fang Xing, Min Yan

**Affiliations:** 1 Department of Anesthesiology, The Second Affiliated Hospital, School of Medicine Zhejiang University, Hangzhou, China; 2 Department of Anesthesiology, Hangzhou Women’s Hospital (The Affiliated Women’s Hospital of Hangzhou Normal University), Hangzhou, China; 3 Department of Anesthesiology, Zhejiang Provincial People’s Hospital (People’s Hospital of Hangzhou Medicine College), Hangzhou, China; 4 Department of Anesthesiology, Women’s Hospital, School of Medicine Zhejiang University, Hangzhou, China

**Keywords:** Tranexamic acid, Fibrinolysis, Tissue plasminogen activator, Cardiac surger

## Abstract

**Background:**

The blood saving efficacy of TXA in cardiac surgery has been proved in several studies, but TXA dosing regimens were varied in those studies. Therefore, we performed this study to investigate if there is a dose dependent in-vivo effect of TXA on fibrinolysis parameters by measurement of fibrinolysis markers in adults undergoing cardiac surgery with CPB.

**Methods:**

A double-blind, randomized, controlled prospective trial was conducted from February 11, 2017 to May 05, 2017. Thirty patients undergoing cardiac valve surgery were identified and randomly divided into a placebo group, low-dose group and high-dose group by 1: 1: 1. Fibrinolysis parameters were measured by plasma levels of D-Dimers, plasminogen activator inhibitor-1 (PAI-1), thrombin activatable fibrinolysis inhibitor (TAFI), plasmin-antiplasmin complex (PAP), tissue plasminogen activator (tPA) and thrombomodulin (TM). Those proteins were measured at five different sample times: preoperatively before the TXA injection (T_1_), 5 min after the TXA bolus (T_2_), 5 min after the initiation of CPB (T_3_), 5 min before the end of CPB (T_4_) and 5 min after the protamine administration (T_5_). A Thrombelastography (TEG) and standard coagulation test were also performed.

**Results:**

Compared with the control group, the level of the D-Dimers decreased in the low-dose and high-dose groups when the patients arrived at the ICU and on the first postoperative morning. Over time, the concentrations of PAI-1, TAFI, and TM, but not PAP and tPA, showed significant differences between the three groups (*P* <  0.05). Compared with the placebo group, the plasma concentrations of PAI-1 and TAFI decreased significantly at the T3 and T4 (*P* <  0.05); TAFI concentrations also decreased at the T5 in low-dose group (*P* < 0.05). Compared with the low-dose group, the concentration of TM increased significantly at the T4 in high-dose group.

**Conclusions:**

The in-vivo effect of low dose TXA is equivalent to high dose TXA on fibrinolysis parameters in adults with a low bleeding risk undergoing valvular cardiac surgery with cardiopulmonary bypass, and a low dose TXA regimen might be equivalent to high dose TXA for those patients.

**Trial registration:**

ChiCTR-IPR-17010303, Principal investigator: Zhen-feng ZHOU, Date of registration: January 1, 2017.

**Supplementary Information:**

The online version contains supplementary material available at 10.1186/s12871-021-01234-8.

## Key points summary

### Question

TXA dosing regimens vary in adults undergoing cardiac surgery, but the in-vivo effect of two tranexamic acid doses on fibrinolysis parameters has not been systematically elucidated.

### Findings

The in-vivo effect of low dose TXA is equivalent to high dose TXA on fibrinolysis parameters in adults undergoing valvular cardiac surgery with cardiopulmonary bypass.

### Meaning

A low dose TXA regimen might be equivalent to high dose TXA for those patients.

## Background

Perioperative bleeding induced by cardiopulmonary bypass (CPB) is a major complication of cardiac surgery [1]. Various factors contribute to CPB-induced coagulation dysfunction, and hyperactivation of fibrinolysis is one of the important causes [2]. Aminocaprioc acid is the most commonly used antifibrinolytic agent in north America and its use recommendation is class Ia [3]. Tranexamic acid (TXA) is used most commonly in Asia due to its cost, wide availability and heat stability despite having slightly lower safety profile than aminocaproid acid [3]. The blood saving efficacy of TXA in cardiac surgery has been proved in several studies [4–6], but the TXA dosing regimens vary in those studies.

The most commonly used TXA dosing regimens are the low-dose regimen reported by Horrow et al [7] and higher-dose regimen described by Dowd et al. [8] Horrow et al. [7] proposed a small-dose dosing regimen according to one early pharmacokinetic study [9] that confirmed TXA concentrations of 10 μg/ml could provide effective inhibition of fibrinolysis. On the other hand, Dowd et al. [8] believed that TXA concentrations should be greater than 126 μg/ml to achieve 100% inhibition of fibrinolysis, and they proposed a high-dose dosing regimen. A recent systematic review concluded that TXA concentrations of 10–15 mg/l could lead to a substantial inhibition fibrinolysis [10], but of the 21 included studies, 20 were in vitro and only one was in vivo. This vivo study only enrolled children undergoing cardiac surgery with either continuous or discontinuous TXA schemes [11]. The dose of TXA is potentially important because TXA also dose-dependently increases seizure risk [12].

A randomized, controlled, prospective trial showed that TXA suppressed fibrinolysis by inhibiting tissue plasminogen activator (tPA) and plasmin activity, however, TXA did not affect other important fibrinolytic inhibitors, such as plasminogen activator inhibitor-1 (PAI-1) and α_2_-antiplasmin [13]. According to the results of an in-vitro study, TXA significantly reduces the clot lysis index (CLI) through tPA-induced hyperfibrinolysis in human whole blood, as analyzed using rotational thromboelastography (TEG) [14].

TXA dosing regimens vary in adults undergoing cardiac surgery, but the in-vivo effect of two tranexamic acid doses on fibrinolysis parameters has not been systematically elucidated. Therefore we performed this feasibility pilot study to investigate if the in-vivo effect of low dose TXA is equivalent to high dose TXA on fibrinolysis parameters by measurement of fibrinolysis markers in adults undergoing valvular cardiac surgery with cardiopulmonary bypass.

## Methods

### Study design and ethics statement

This study was approved by The Ethics Committee of Second Affiliated Hospital of Zhejiang University (IRB:2016–049) and written informed consent was obtained from all subjects participating in the trial. Study protocol was presented as supporting information and the trial was registered prior to patient enrollment at clinicaltrials.gov (ChiCTR-IPR-17010303). A double-blind, randomized, and controlled prospective trial was conducted from February 11, 2017 to May 05, 2017.

### Inclusion and exclusion criteria

Inclusion criteria were patients undergoing elective valvular cardiac surgery with CPB and an age greater than 18 years. Exclusion criteria were a history of cerebral infarction, the presence of arterial or venous thrombosis, a history of myocardial infarction in the previous 7 days, preoperative chronic kidney disease [CKD] (serum creatinine (Cr) by 1.6 mg/dl for men and > 1.4 mg/dl for women or needing for renal replacement therapy), preoperative chronic liver disease (grade B or C of the Child-Pugh classification), a previous history of endocarditis, anemia(< 120 g/dl for men and < 110 g/dl for women), hyperlipidemia, acute heart failure, preoperative shock, treatment with preoperative coagulation medication within 5 days of surgery (warfarin, aspirin, antifibrinolytic or thrombolytic treatment), preoperative coagulopathy (international normalized ratio (INR) > 1.5, platelet count < 100 × 10^3^/mm^3^, fibrinogen < 1 g/L), previous sternotomy, emergency procedures, endocarditis, combined procedures (combined with coronary artery bypass graft surgery, aortic surgery, carotid surgery, other nonvalvular surgery, experienced deep hypothermic circulatory arrest), allergy or contraindication to tranexamic acid, pregnancy, and participation in another study.

### Randomisation and procedures

Thirty patients were identified and randomly divided into a placebo group, low-dose TXA group and high-dose TXA group by 1:1:1 using numbered sealed envelopes. Patients, surgical team, and data investigators were unaware of the group assignments. Intra-operative TXA (Conba Bio-Pharm.Co.,Ltd., Jin-hua Zhe-jiang, 0.5 g/100 ml) was injected into the central vein by the anesthesiologist until the wound dressings were placed.

The low-dose scheme was adapted from Horrow et al. [7] and the high-dose method was based on the dosing regimen reported by Dowd et al. [8] In the low-dose TXA group, patients received a loading dose of 10 mg/kg 15 min after intubation, followed by a 1 mg/kg/h infusion. In the high-dose TXA group, a 30 mg/kg bolus was administered, followed by continuous infusion of 16 mg/kg/h. Additional TXA doses of 1 mg/kg and 2 mg/kg were added to the venous reservoir of the low-dose and high-dose groups, respectively, during CPB.

An independent investigator generated the random allocation sequence and informed another independent nurse who prepared the study drug with a 100 ml isotonic solution for bolus administration (TXA concentration: low dose = 0.1 mg/kg/ml; high dose = 0.3 mg/kg/ml) 10 min before administration of TXA. A 50 ml isotonic solution was continuously infused at a rate of 5 ml/h during the operation (TXA concentration: low dose = 0.2 mg/kg/ml; high dose = 3.2 mg/kg/ml) and a 20 ml syringe contained the priming solution in the venous reservoir (TXA concentration: low dose = 0.05 mg/kg/ml; high dose = 0.1 mg/kg/ml). It was prepared only with isotonic solution in the placebo group. Other perioperative anesthesia management, blood transfusions strategy and post-operative management strategy was consistent with our previous published study [15].

Blood samples were collected into tubes containing 3.8% sodium citrate from the internal jugular vein at the following sample points: (T1) preoperatively before TXA injection (baseline), (T2) 5 min after the administration of the TXA bolus (bolus), (T3) 5 min after the onset of CPB (CPB), (T4) 5 min before the end of CPB (end of CPB) and (T5) 5 min after the protamine injection (protamine). The tube was immediately placed in a 4 °C refrigerator, centrifuged for 20 min at 3000 rpm at 4 °C within 1 h, and then stored at − 80 °C until further testing.

### Primary outcome

The intraoperative plasma levels of tissue plasminogen activator (tPA).

### Secondary outcome

1. The intraoperative plasma levels of other coagulation proteins as following: plasminogen activator inhibitor-1 (PAI-1), thrombin activatable fibrinolysis inhibitor (TAFI),plasmin-antiplasmin complex (PAP), tissue plasminogen activator (tPA), and thrombomodulin (TM). An independent investigator assayed the plasma concentrations of those coagulation proteins with enzyme immunoassays according to the manufacturer’s instructions (ELISA; Shanghai Jianglai Biotech, Shanghai, China). The details of those coagulation proteins are presented in supplemental Table 1.

The changes in the concentrations of coagulation proteins caused by hemodilution were corrected using the following formula: corrected concentration = (sample blood concentration × baseline blood hematocrit)/sample blood hematocrit [16].

2. The Perioperative Fibrinogen, D-Dimers, standard coagulation test and TEG test.

3. Postoperative clinical data when arriving at ICU and in the first post-operative morning.

### Sample size calculation

One-way analysis of variance (ANOVA) was applied and the type I error was assumed by 0.05, we estimated that 7 patients were required for each group to provide 95% power to detect an effect of different doses of TXA on successively changing 33% of the activity of the fibrinolytic system when the standard deviation of coagulation proteins concentration was limited to within 20% [17]. A target sample size of 30 was chosen based in this pilot investigation.

### Statistical methods

The variables with a normal distribution are reported as means ± standard deviations (SD), and continuous variables with a non-normal distribution are reported as medians (interquartile ranges). ANOVA was used to compare continuous variables with a normal distribution between the groups, and Welch’s test was adopted when variance existed between the groups and the different time points. Analysis of variance for repeated measurements was applied for repeated measured variables between the groups and the different times. The homogeneity of variance was analyzed using Mauchly’s test of sphericity and corrected with the Greenhouse-Geisser test if variance existed. Differences in the concentrations of coagulation proteins between the groups measured at the same time points were analyzed using two-way ANOVA followed by the Bonferroni test. The Kruskal-Wallis H test was applied to continuous variables with a non-normal distribution. The Chi-squared or Fisher’s exact test was used to analyze categorical data. All reported *P* values were two sided, and *P* values less than 0.05 were considered significant. Missing data was less than 10% and was not replaced. Intention to treat analysis was used and analysis was performed with SPSS version 18 (IBM, Armonk, NY) and G-Power (version 3.1; Informer Technologies, Inc.).

## Results

### Baseline parameters and operative characteristics

**Fig. 1:**
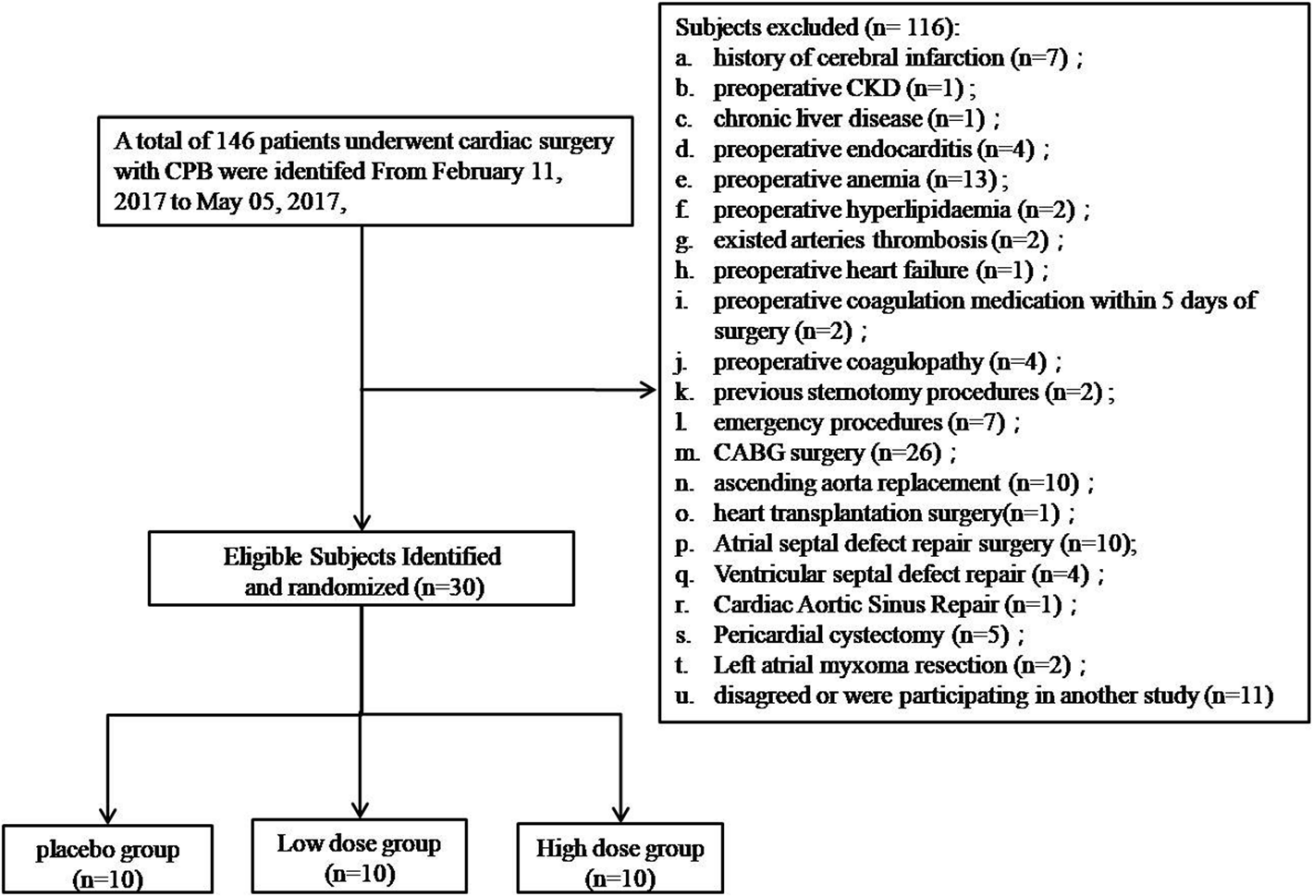
Study flowchart Study population recruitment summary

Of 146 patients who underwent cardiac surgery with CPB, 30 patients participated and completed the follow- up of this study, and 116 patients met the exclusion criteria as shown in Fig. 1. The basic clinical features of the three groups are presented in Supplemental Table 2 and no difference was observed.

The average age of the participations was 58 years, 60% were female, 93.3% had ASA (American Society of Anesthesiologists) class III and the other patients had ASA grade IV, 20% had NYHA (New York Heart Association) grade III and 6.7% had grade IV. The average score of the European Cardiovascular Surgery Risk Factor Scoring System (EuroSCORE) was 1.6, and the average score for the bleeding risk was only 0.8. 36.7% of patients underwent aortic valve surgery, 23.3% underwent mitral valve surgery, 36.7% underwent surgery on multiple valves, and only one patient underwent simple tricuspid valve surgery.

We did not observe any side effects of TXA in those patients during the study period, such as stroke, death and re-operation for bleeding.

### Primary outcome

Over time, there was no significant difference in the plasma concentrations with correcting for hemodilution of tPA between the three groups (Table 1), however, the plasma concentrations without correcting for hemodilution of tPA showed significant differences between the three groups (*P* = 0.018) (Supplemental Table 3).

**Table 1:** Coagulation proteins with correcting for hemodilution at different time points between the three groups

	Placebo group (*n* = 10)	Low dose group (*n* = 10)	High dose group (*n* = 10)	*P*-value
PAI-1 [mean (SD); ng/mL]				0.032
T_1_	23.7 ± 2.5	22.9 ± 2.5	24.1 ± 2.2	
T_2_	28.2 ± 1.9	27.6 ± 2.8	28.4 ± 1.9	
T_3_	56 ± 5	50 ± 7^a^	57 ± 6	
T_4_	52 ± 6	45 ± 8^a^	51 ± 6	
T_5_	43.0 ± 2.2	39.6 ± 7.9	43.2 ± 4.5	
TAFI [mean (SD); ng/mL]				0.046
T_1_	17.5 ± 1.6	18.1 ± 1.1	16.4 ± 0.7	
T_2_	20.9 ± 1.7	21.8 ± 2.0	21.5 ± 1.6	
T_3_	43.0 ± 2.9	37.1 ± 4.4^a^	40.5 ± 5.2	
T_4_	37 ± 5	32 ± 6^a^	37 ± 5	
T_5_	33.5 ± 2.2	29.4 ± 4.1^a^	32.5 ± 3.6	
PAP [mean (SD); ng/mL]				0.176
T_1_	13.9 ± 1.9	15.8 ± 1.5	13.3 ± 2.1	
T_2_	19.7 ± 2.4	20.2 ± 2.3	19.7 ± 2.5	
T_3_	42 ± 4	39 ± 6	45 ± 8	
T_4_	37 ± 7	34 ± 7	39 ± 6	
T_5_	31 ± 6	28 ± 3	33 ± 5	
tPA [mean (SD); ng/mL]				0.113
T_1_	3.0 ± 0.3	3.5 ± 0.4	3.3 ± 0.6	
T_2_	3.67 ± 0.37	4.24 ± 0.55	4.17 ± 0.24	
T_3_	7.9 ± 1.2	7.5 ± 1.6	8.3 ± 1.1	
T_4_	6.6 ± 1.1	6.3 ± 1.0	7.2 ± 1.1	
T_5_	5.7 ± 1.1	5.6 ± 0.7	6.3 ± 0.9	
TM [mean (SD); ng/mL]				0.019
T_1_	1.33 ± 0.09	1.55 ± 0.15	1.57 ± 0.14	
T_2_	1.69 ± 0.16	1.65 ± 0.15	1.72 ± 0.18	
T_3_	3.5 ± 0.5	3.2 ± 0.5	3.7 ± 0.6	
T_4_	2.9 ± 0.4	2.6 ± 0.5	3.3 ± 0.7^b^	
T_5_	2.66 ± 0.48	2.24 ± 0.32	2.57 ± 0.28	

^a^: Low-dose group compared with placebo group;^b^: High-dose group compared with Low-dose group. *PAI-1* Plasminogen activator inhibitor-1, *TAFI* Thrombin activatable fibrinolysis inhibitor, *PAP* Plasmin-antiplasmin complex, *tPA* Tissue plasminogen activator, *TM* Thrombomodulin. T_1_: per-operatively before TXA injection (baseline); T_2_: 5 min after TXA bolus administration (bolus); T_3_: 5 min after the onset of CPB (CPB); T_4_: 5 min before the end of CPB (End of CPB); T_5_: 5 min after protamine injection (protamine)

### Secondary outcome

#### Intraoperative levels of other coagulation proteins

Over time, the plasma concentrations with correcting for hemodilution of PAI-1(*P* = 0.032), TAFI (*P* = 0.046), and TM (*P* = 0.019), but not PAP, showed significant differences between the three groups (Table 1). However, the plasma concentrations without correcting for hemodilution of them showed no significant difference between the three groups (Supplemental Table 3).

**Fig. 2:**
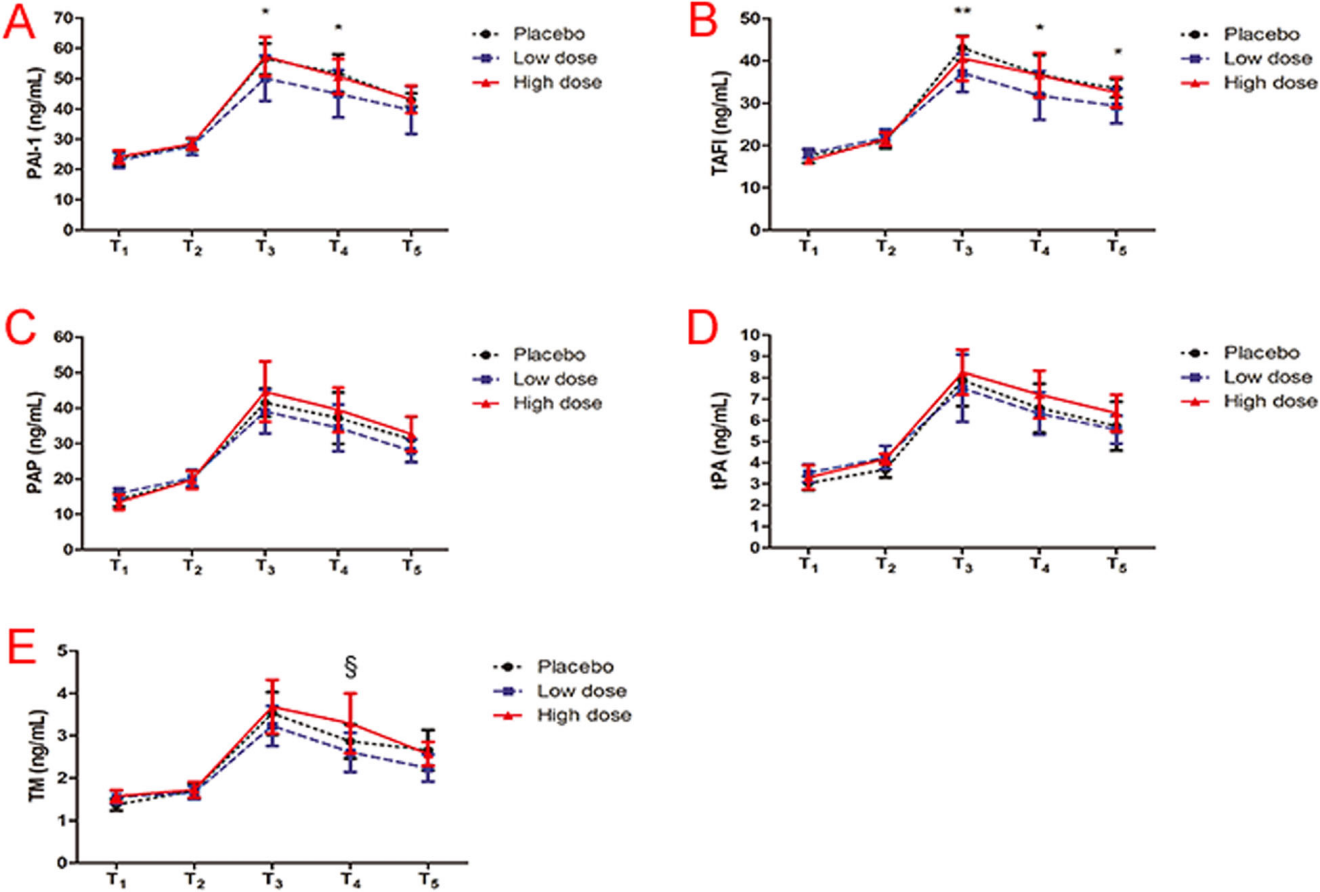
Concentration curves of Coagulation proteins at different sample points between the three groups. PAI-1: plasminogen activator inhibitor-1, TAFI: thrombin activatable fibrinolysis inhibitor, PAP: plasmin-antiplasmin complex, tPA: tissue plasminogen activator, TM: thrombomodulin. T_1_: per-operatively before TXA injection (baseline); T_2_: 5 min after TXA bolus administration (bolus); T_3_: 5 min after the onset of CPB (CPB); T_4_: 5 min before the end of CPB (End of CPB); T_5_: 5 min after protamine injection (protamine). *: Low-dose group compared with placebo group; ^§^: High-dose group compared with Low-dose group

Compared with the placebo group, the plasma concentrations of PAI-1 (Fig. 2a) and TAFI (Fig. 2b) decreased significantly at the T3 and T4 sample points (*P* < 0.05); TAFI concentrations also decreased at the T5 sample point in the low-dose group (*P* < 0.05). No significant differences were observed in the levels of the coagulation proteins at any other sample points between the three groups (Fig. 2c for PAP and Fig. 2d for tPA). Compared with the low-dose group, the plasma concentration of TM (Fig. 2e) increased significantly at the T4 sample point in the high-dose group (*P* < 0.05) (Table 1).

#### Perioperative fibrinogen, D-dimers and TEG test

Compared with the control group, the level of the D-Dimers decreased in the low-dose and high-dose groups when the patients arrived at the ICU (*P* < 0.001) and on the first postoperative morning (*P* = 0.008), but no differences were observed in other measurement points. Compared with the control group, fibrinogen measured on the first post-operative morning was higher in high-dose groups (*P* = 0.027), but no differences were observed in other measurement points. Significant differences in intra-operative standard coagulation tests and TEG analysis were not observed (Table 2 and Supplemental Table 4).

**Table 2:** Perioperative Fibrinogen, D-Dimers and TEG test at different time points between the three groups

	Placebo group (*n* = 10)	Low dose group (*n* = 10)	High dose group (*n* = 10)	*P*-value
**Fibrinogen [mean (SD); g/L]**				
Preoperative	3.2 ± 0.9	3.0 ± 0.4	3.5 ± 1.1	0.408
Intra-operative period				0.786
T_1_	3.1 ± 0.8	3.1 ± 0.4	3.4 ± 0.8	
T_2_	3.0 ± 0.9	2.9 ± 0.4	3.2 ± 0.8	
T_3_	1.43 ± 0.30	1.55 ± 0.25	1.50 ± 0.35	
T_4_	1.7 ± 0.4	1.8 ± 0.4	1.7 ± 0.5	
T_5_	1.7 ± 0.5	1.9 ± 0.4	1.8 ± 0.5	
when arriving at ICU	2.0 ± 0.5	2.1 ± 0.4	2.1 ± 0.5	0.875
In the first post-operative morning	2.9 ± 1.1	3.1 ± 1.2	4.3 ± 0.8^b^	0.027
**D-Dimer [median (IQR); ug/L]**				
Preoperative	290 (213–705)	235 (188–518)	280 (210–665)	0.715
Intra-operative period				0.465
T_1_	305 (255–470)	305 (228–445)	295 (220–500)	
T_2_	330 (270–490)	315 (270–443)	295 (220–545)	
T_3_	305 (248–370)	220 (220–270)	235 (220–308)	
	750 (415–1133)	420 (318–555)	280 (220–393)	
T_5_	745 (430–1343)	480 (235–605)	255 (220–373)	
when arriving at ICU	1540 (1078–1900)	555 (483–770)^a^	260 (220–440)^b^	< 0.001
In the first post-operative morning	1530 (1030–2155)	710 (520–1070)^a^	540 (405–880)^b^	0.008
TEG-R [mean (SD); min]				0.439
T_1_	6.8 ± 1.7	5.9 ± 1.1	5.7 ± 1.0	
T_2_	6.3 ± 1.9	6.4 ± 1.4	5.5 ± 0.8	
T_5_	5.9 ± 1.5	7.9 ± 2.7	6.7 ± 3.2	
TEG-K [median (IQR); min]				0.551
T_1_	1.75 (1.68–2.50)	1.60 (1.40–1.85)	1.35 (1.15–1.63)	
T_2_	2.00 (1.30–2.45)	1.85 (1.55–1.93)	1.65 (1.20–2.05)	
T_5_	2.40 (1.85–3.05)	2.30 (2.00–3.10)	2.60 (1.85–3.20)	
TEG-Angle [mean (SD); °]				0.420
T_1_	68.8 ± 7.0	72.1 ± 3.3	74.3 ± 2.3	
T_2_	70 ± 5	68 ± 9	73 ± 4	
T_5_	68 ± 5	65 ± 8	66 ± 7	
TEG-MA [mean (SD); mm]				0.618
T_1_	63 ± 6	64 ± 3	66 ± 4	
T_2_	62 ± 5	63 ± 4	66 ± 4	
T_5_	52 ± 6	52 ± 5	51 ± 9	
TEG-EPL [median (IQR); %]				0.779
T_1_	0.20 (0–0.90)	0 (0–0.18)	0 (0–0.38)	
T_2_	0 (0–0.20)	0 (0–0.03)	0 (0–0.10)	
T_5_	0 (0–0.80)	0 (0–0.45)	0 (0–0.85)	
TEG-A [mean (SD); mm]				0.600
T_1_	62 ± 6	63 ± 4	65 ± 6	
T_2_	62 ± 5	62 ± 5	65 ± 6	
T_5_	50 ± 6	51 ± 7	51 ± 10	
TEG-LY30[median (IQR); %]				0.427
T_1_	0 (0–0.25)	0 (0–0.13)	0 (0–0.23)	
T_2_	0 (0–0.05)	0 (0–0.03)	0 (0–0.10)	
T_5_	0 (0–0.8)	0 (0–0.45)	0 (0–0.85)	
TEG-CI [median (IQR)]				0.433
T_1_	0.8(−1.2–1.2)	1.0 (0.5–1.7)	1.8 (1.3–2.6)	
T_2_	−0.2[(−1.0)-2.2]	0.9[(−0.6)-1.7]	1.4 (0.6–2.4)	
T_5_	−0.9[(−2.5)-(−0.2)]	−2.1[(−5.2)-(−0.4)]	−1.5[(−2.9)-0.7]	

^a^: Low-dose group compared with placebo group; ^b^: High-dose group compared with placebo group. T_1_ = per-operatively before TXA injection (baseline); T_2_ = 5 min after TXA bolus administration (bolus); T_3_ = 5 min after the onset of CPB (CPB); T_4_ = 5 min before the end of CPB (End of CPB); T_5_ = 5 min after protamine injection (protamine)

T_1_ = per-operatively before TXA injection (baseline); T_2_ = 5 min after TXA bolus administration (bolus); T_3_ = 5 min after the onset of CPB (CPB); T_4_ = 5 min before the end of CPB (End of CPB); T_5_ = 5 min after protamine injection (protamine). *R* Reaction time, *MA* Maximum amplitude, *A* Amplitude of clot firmness after clotting time, *CI* Coagulation index

Intra-operative levels of coagulation proteins involved in thrombin generation and the fibrinolytic response.

#### Postoperative clinical data when arriving at ICU and in the first post-operative morning

No significant differences were observed in clinical data and standard coagulation test on the first postoperative morning (Supplemental Table 5).

## Discussion

In this study, we found that the vivo effect of low dose TXA is equivalent to high dose TXA on fibrinolysis parameters in adults undergoing valvular cardiac surgery with cardiopulmonary bypass with a low bleeding risk. Our study support previous studies [4, 6], which showed that low dose TXA is equivalent to high dose TXA in efficacy. Furthermore, the increasing TM produces in the high dose TXA regimen, which raises the potential concern on the safety of high-dose TXA.

Hemodilution tends to obscure the underlying changes occurring in the hemostatic system during CPB [2]. All factors in blood including coagulation factors were diluted equally immediately for hemodilution by the priming fluid in the bypass circuit at the start of CPB [2], so those factors must be undergoing a rapid increase in formation to maintain its concentration at preoperative levels. With this kind of data, it is difficult to gauge what the underlying rate of activation is. One approach is correction for hemodilution, that is, dividing results by the average percent change in stable factor levels to give an indication of what the marker level would be if hemodilution had not occurred [2].

Compared with the placebo group, we found that the D-dimer level, was significantly decreased in patients receiving both the two TXA doses upon their arrival at the ICU and on the first postoperative morning. We also noticed that although no difference of D-dimer was found between the two TXA doses, it seemed that there was a dose dependent effect of TXA on D-dimer. Although fibrinogen measured on the first post-operative morning was increased in high-dose groups as compared to control group, no difference was found between the two TXA doses. We speculated that TXA would not achieve greater inhibition of fibrinolytic activity when TXA exceeds a certain plasma concentration. This finding may support the hypothesis proposed in a previous study of a platform effect of TXA on inhibiting fibrinolysis [18], and a higher TXA dose should be used with caution in clinical practice.

This small sample study did not observe dose-dependent changes in the levels of tPA and PAP between groups treated with the two TXA doses regimens. According to another previous study, aminocaproic acid does not inhibit the formation of active plasmin and reduce PAP levels [19], and the peak PAP concentration was detected during the postoperative period [17]. The concentration of tPA increased immediately after CPB began and continued to increase throughout the entire surgical period. However, tPA levels were similar in the groups treated with the two TXA doses and placebo. A previous study examining the kinetics of aprotinin-mediated inhibition of plasmin reported similar results showing similar tPA levels in the aprotinin and placebo groups [20]. However, the authors noticed that tPA concentrations mainly increased during CPB, but PAI-1 concentrations gradually increased after CPB, which is inconsistent with the findings of our study [20]. We noticed that the levels of proteins that promote (tPA and PAP) and inhibit fibrinolysis (PAI-1 and TAFI) were both increased 5 min after CPB began, and then they reached new balanced levels. This discrepancy might due to the analysis of participants with a low bleeding risk and a relatively low degree of intra-operative fibrinolytic activity. Therefore, the participants with a pre-operative normal coagulation function likely readily reached a new fibrinolytic balance.

Many possible factors may also explain the different results. First, although the active tPA and plasmin levels increase approximately 5-fold during CPB, approximately one third of patients might not show any change [21]. Second, the effect of TXA is mediated by many proteins involved in the coagulation process including kallikrein, activated protein C, and thrombin [22]; therefore, different TXA doses may reduce bleeding through different mechanisms. Third, the difference also might due to a relatively low degree of fibrinolytic activity during CPB in adults with a low bleeding risk who are undergoing cardiac valvular surgery. Compared with the levels measured before CPB, the concentrations of tPA and other coagulation proteins during CPB were approximately 1.5 times higher in this study, which differ from a previous study reporting that the active tPA concentration increases approximately 5 times [23]. This effect might be due to the improved compatibility of CPB materials and improved CPB and surgical technologies, which would minimize fibrinolytic activation during CPB, thereby preventing the excessive consumption of coagulation proteins, fibrinogen, and platelets. We speculated that the effect of intraoperative TXA-mediated tPA inhibition on fibrinolysis may be overestimated, and this effect on inhibiting fibrinolytic activity may occur after protamine antagonism or during the postoperative period.

A randomized, controlled, prospective trial showed that TXA (100 mg/kg) suppressed fibrinolysis by inhibiting tissue plasminogen activator (tPA) and plasmin activity, however, TXA did not affect other important fibrinolytic inhibitors, such as plasminogen activator inhibitor-1 (PAI-1) and α_2_-antiplasmin [13]. Similar to the changes in tPA levels, PAI-1 levels do not change in approximately one-third of patients after surgery [24]. PAI-1 prevents plasmin formation. PAI-1 levels increase 15-fold only 2 h after surgery [25], and the levels are maintained until the first postoperative day.

TXA preserves the size and strength of the thrombus in a rat model of a simulated arterial aneurysm [26]. So we also measured TM, a marker of vascular endothelial function [27], increases in TM in patients with coronary atherosclerosis [28]. Compared with the low TXA group, we found that TM increased significantly at 5 min after CPB in the high-dose group. Thrombotic complications tend to form when endothelial dysfunction exists. TXA increases the risk of thrombus formation in a dose-dependent manner both in vitro and in vivo. This finding differs from aprotinin, which inhibits thrombus formation [29]. TXA not only reduces the carotid artery occlusion time but also increases the stiffness of the thrombus in a rat model of ferric chloride-induced thrombosis [30]. Increased thrombosis has been observed after treatment with different TXA doses (30,100 and 300 mg/kg/h) in vitro and in vivo animal models designed to evaluate fibrinolysis and thrombus formation [31]. Importantly, fibrinolysis decreases after surgery. Furthermore, individual variability in the response to CPB was another reason why the prediction of patients at risk for bleeding or thrombosis is difficult to determine. The increasing TM produces in the high dose TXA regimen in this study, which raises the potential concern on the safety of high-dose TXA.

Our results were consistent with previous studies [18] that standard coagulation test and TEG test did not reflect a difference in the effects of the two TXA doses on inhibiting fibrinolysis in patients undergoing cardiac surgery with CPB. Previous study found that a ROTEM examination could only detect significant fibrinolysis [32].

### Limitations

This study has several limitations. First, the total sample was relatively small. It is impossible to know if the differences reported in term of biomarkers would translate into a clinically meaningful difference in term of bleeding and transfusion, and other outcomes. This pilot investigation study was aimed to compared the in-vivo effect of two TXA doses on fibrinolysis parameters in adults undergoing valvular cardiac surgery and the blood saving efficacy of TXA in cardiac surgery has been proved in several studies [4–6]. As shown in this study, the in-vivo effect of low dose TXA is equivalent to high dose TXA on fibrinolysis parameters and no further improvement was observed for high dose TXA. So we suggested a low dose TXA regimen for those patients with a low bleeding risk. Due to low incidence and small sample, we did not observe the side-effect of TXA. We also did not do related neurological examination and questionnaires. Second, the plasma TXA concentration was not monitored at different sample points during the study. However, many previous studies have investigated plasma TXA concentrations in patients treated with different TXA doses [33]. Third, this study evaluated postoperative coagulation function only by performing a standard coagulation test and failed to monitor postoperative levels of coagulation proteins, which require further exploration in a future study.

## Conclusions

The in-vivo effect of low dose TXA is equivalent to high dose TXA on fibrinolysis parameters in adults with a low bleeding risk undergoing valvular cardiac surgery with cardiopulmonary bypass, and a low dose TXA regimen might be equivalent to high dose TXA for those patients. Furthermore, the increasing TM produces in the high dose TXA regimen, which raises the potential concern on the safety of high-dose TXA. Further large and multicenter studies are needed to confirm the clinical impact of different doses TXA for those patients.

## Supplementary Information

**Additional file 1: Table S1.** Coagulation proteins of fibrinolysis parameters.

**Additional file 2: Table S2.** Demographic and surgical data.

**Additional file 3: TableS3.** Coagulation proteins without correcting for hemodilution at different time points between the three groups.

**Additional file 4: TableS4.** Platelet counts, standard coagulation test and TEG test at different time points between the three groups.

**Additional file 5: TableS5.** Postoperative clinical data and Standard coagulation test.

## Data Availability

The datasets used during the current study available from the corresponding author on reasonable request.

## Abbreviations

ANH: Acute Normovolemic Hemodilution
ANOVA: One-way analysis of variance
ASA: American Standards Association
CKD: Chronic kidney disease
CLI: Clot lysis index
CPB: Cardiopulmonary Bypass
Cr: Serum creatinine
EuroSCORE: European Cardiovascular Surgery Risk Factor Scoring System
ICU: Intensive Care Unit
INR: International normalized ratio
NYHA: New York Heart Association
PAI-1: Plasminogen activator inhibitor-1
PAP: Plasmin-antiplasmin complex
SD: Standard deviations
TAFI: Thrombin activatable fibrinolysis inhibitor
TM: Thrombomodulin
TEG: Thromboelastography
tPA: Tissue plasminogen activator
TXA: Tranexamic acid

## References

[1] Hessel EA (2019). What's new in cardiopulmonary bypass. J Cardiothorac Vasc Anesth.

[2] Sniecinski RM, Chandler WL (2011). Activation of the hemostatic system during cardiopulmonary bypass. Anesth Analg.

[3] Ferraris VA, Brown JR, Society of Thoracic Surgeons Blood Conservation Guideline Task Force (2011). 2011 update to the Society of Thoracic Surgeons and the Society of Cardiovascular Anesthesiologists blood conservation clinical practice guidelines. Ann Thorac Surg.

[4] Sigaut S, Tremey B, Ouattara A (2014). Comparison of two doses of tranexamic acid in adults undergoing cardiac surgery with cardiopulmonary bypass. Anesthesiology..

[5] Myles PS, Smith JA, Forbes A (2017). Tranexamic acid in patients undergoing coronary-artery surgery. N Engl J Med.

[6] Du Y, Xu J, Wang G (2014). Comparison of two tranexamic acid dose regimens in patients undergoing cardiac valve surgery. J Cardiothorac Vasc Anesth.

[7] Horrow JC, Van Riper DF, Strong MD, Grunewald KE, Parmet JL (1995). The dose-response relationship of tranexamic acid. Anesthesiology..

[8] Dowd NP, Karski JM, Cheng DC (2002). Pharmacokinetics of tranexamic acid during cardiopulmonary bypass. Anesthesiology..

[9] Andersson L, Nilsoon IM, Colleen S, Granstrand B, Melander B (1968). Role of urokinase and tissue activator in sustaining bleeding and the management thereof with EACA and AMCA. Ann N Y Acad Sci.

[10] Picetti R, Shakur-Still H, Medcalf RL, Standing JF, Roberts I (2019). What concentration of tranexamic acid is needed to inhibit fibrinolysis? A systematic review of pharmacodynamics studies. Blood Coagul Fibrinolysis.

[11] Couturier R, Rubatti M, Credico C (2014). Continuous or discontinuous tranexamic acid effectively inhibits fibrinolysis in children undergoing cardiac surgery with cardiopulmonary bypass. Blood Coagul Fibrinolysis.

[12] Murkin JM, Falter F, Granton J, Young B, Burt C, Chu M (2010). High-dose tranexamic acid is associated with nonischemic clinical seizures in cardiac surgical patients. Anesth Analg.

[13] Kojima T, Gando S, Morimoto Y (2001). Systematic elucidation of effects of tranexamic acid on fibrinolysis and bleeding during and after cardiopulmonary bypass surgery. Thromb Res.

[14] Dirkmann D, Görlinger K, Gisbertz C, Dusse F, Peters J (2012). Factor XIII and tranexamic acid but not recombinant factor VIIa attenuate tissue plasminogen activator-induced hyperfibrinolysis in human whole blood. Anesth Analg.

[15] Zhou ZF, Zhang FJ, Huo YF (2017). Intraoperative tranexamic acid is associated with postoperative stroke in patients undergoing cardiac surgery. PLoS One.

[16] Welters I, Menges T, Ballesteros M (1998). Thrombin generation and activation of the thrombomodulin protein C system in open heart surgery depend on the underlying cardiac disease. Thromb Res.

[17] Kuitunen A, Hiippala S, Vahtera E, Rasi V, Salmenperä M (2005). The effects of aprotinin and tranexamic acid on thrombin generation and fibrinolytic response after cardiac surgery. Acta Anaesthesiol Scand.

[18] Faraoni D, Cacheux C, Van Aelbrouck C, Ickx BE, Barvais L, Levy JH (2014). Effect of two doses of tranexamic acid on fibrinolysis evaluated by thromboelastography during cardiac surgery: a randomised, controlled study. Eur J Anaesthesiol.

[19] de Bono DP, Pringle S, Underwood I (1991). Differential effects of aprotinin and tranexamic acid on cerebral bleeding and cutaneous bleeding time during rt-PA infusion. Thromb Res.

[20] Kang HM, Kalnoski MH, Frederick M, Chandler WL (2005). The kinetics of plasmin inhibition by aprotinin in vivo. Thromb Res.

[21] Tanaka K, Takao M, Yada I, Yuasa H, Kusagawa M, Deguchi K (1989). Alterations in coagulation and fibrinolysis associated with cardiopulmonary bypass during open heart surgery. J Cardiothorac Anesth.

[22] Levy JH, Sypniewski E (2004). Aprotinin: a pharmacologic overview. Orthopedics..

[23] Chandler WL, Velan T (2003). Secretion of tissue plasminogen activator and plasminogen activator inhibitor 1 during cardiopulmonary bypass. Thromb Res.

[24] Chandler WL, Fitch JC, Wall MH (1995). Individual variations in the fibrinolytic response during and after cardiopulmonary bypass. Thromb Haemost.

[25] Chandler WL, Velan T (2004). Plasmin generation and D-dimer formation during cardiopulmonary bypass. Blood Coagul Fibrinolysis.

[26] Patterson RH, Harpel P (1971). The effect of epsilon aminocaproic acid and tranexamic acid on thrombus size and strength in a simulated arterial aneurysm. J Neurosurg.

[27] Lisowska A, Lisowski P, Knapp M (2014). Serum adiponectin and markers of endothelial dysfunction in stable angina pectoris patients undergoing coronary artery bypass grafting (CABG). Adv Med Sci.

[28] Merlini PA, Rossi ML, Faioni EM (2004). Expression of endothelial protein C receptor and thrombomodulin in human coronary atherosclerotic plaques. Ital Heart J.

[29] McCormack PL (2012). Tranexamic acid: a review of its use in the treatment of hyperfibrinolysis. Drugs..

[30] Lockyer S, Kambayashi J (1999). Demonstration of flow and platelet dependency in a ferric chloride-induced model of thrombosis. J Cardiovasc Pharmacol.

[31] Sperzel M, Huetter J (2007). Evaluation of aprotinin and tranexamic acid in different in vitro and in vivo models of fibrinolysis, coagulation and thrombus formation. J Thromb Haemost.

[32] Raza I, Davenport R, Rourke C (2013). The incidence and magnitude of fibrinolytic activation in trauma patients. J Thromb Haemost.

[33] Grassin-Delyle S, Tremey B, Abe E (2013). Population pharmacokinetics of tranexamic acid in adults undergoing cardiac surgery with cardiopulmonary bypass. Br J Anaesth.

