# Supplementary material for: Comparison of the in-vivo effect of two tranexamic acid doses on fibrinolysis parameters in adults undergoing valvular cardiac surgery with cardiopulmonary bypass - a pilot investigation
Source: BMC Anesthesiol. 2021 Feb 2;21:33. doi: 10.1186/s12871-021-01234-8 (PMC7852217; doi:10.1186/s12871-021-01234-8)
Supplement: Supplementary file 1 — Additional file 1: Table S1. Coagulation proteins of fibrinolysis parameters. [file 12871_2021_1234_MOESM1_ESM.doc]

| Supplemental Table1: Coagulation proteins of fibrinolysis parameters | | | |
| --- | --- | --- | --- |
| Coagulation factors |  | | function |
| PAI-1 |  | Inhibition of tPA amd uPA | |
| TAFI |  | Inhibits the conversion of plasminogen to plasmin | |
| PAP |  | PAP is quickly formed once plasmin is generated and combined with α2-antiplasmin (α2-AP), then PAP can inactivate plasmin. | |
| tPA |  |  | |
| TM |  | TM combined with thrombin to forms a complex, then can transforms protein C into APC (activated protein C) and enhances fibrinolytic activity. | |
| PAI-1= plasminogen activator inhibitor-1; uPA= urokinase plasminogen activators; TAFI= thrombin activatable fibrinolysis inhibitor; PAP= plasmin-antiplasmin complex; tPA= tissue plasminogen activator; TM= Thrombomodulin. | | | |
