# Supplementary material for: Comparison of the in-vivo effect of two tranexamic acid doses on fibrinolysis parameters in adults undergoing valvular cardiac surgery with cardiopulmonary bypass - a pilot investigation
Source: BMC Anesthesiol. 2021 Feb 2;21:33. doi: 10.1186/s12871-021-01234-8 (PMC7852217; doi:10.1186/s12871-021-01234-8)
Supplement: Supplementary file 2 — Additional file 2: Table S2. Demographic and surgical data. [file 12871_2021_1234_MOESM2_ESM.doc]

| Supplemental Table 2. Demographic and surgical data. | | | | | | | | | | |
| --- | --- | --- | --- | --- | --- | --- | --- | --- | --- | --- |
|  | placebo group  (n= 10) | low dose  group (n= 10) | | | high dose  group (n= 10) | | *P*-value | | |  |
| Age [mean(SD); yr] | 59.4 ± 13.9 | 56.1 ± 5.7 | | | 58.4 ± 10.2 | | 0.774 | |  | |
| Male/female, no. (%) | 5/5(50%) | 5/5(50%) | | | 2/8(20%) | | 0.287# | |  | |
| BMI [mean(SD); kg/m^2^] | 22.4 ± 2.1 | 24.4 ± 3.4 | | | 23.0 ± 3.1 | | 0.279 | |  | |
| ASA, III/IV/V, (n) | 9/1/0 | 10/0/0 | | | 9/1/0 | | 0.585# | |  | |
| NYHA class, II/III/IV/V, (n) | 6/2/2 | 8/2/0 | | | 8/2/0 | | 0.359# | |  | |
| EuroSCORE[median(IQR)] | 2.00(0-3.25) | 1.00(0-1.25) | | | 1.00(1.00-2.50) | | 0.365 | |  | |
| LVEF, (n) |  |  | | |  | | 0.722# | |  | |
| > 50%/35-50%/20-34% | 8/1/1 | 9/1/0 | | | 9/1/0 | |  | |  | |
| History of smoking, no. (%) | 5(50%) | | 3(30%) | 1(10%) | | 0.149# | |  | | |
| Coexistent disease, no. (%) |  | |  |  | |  | |  | | |
| AF | 3(30%) | | 3(30%) | 4(40%) | | 0.861# | |  | | |
| Hypertension | 2(20%) | | 6(60%) | 3(30%) | | 0.155# | |  | | |
| Diabetes | 0(0%) | | 0(0%) | 1(10%) | | 0.355# | |  | | |
| Cerebrovascular disease | 0(0%) | | 1(0%) | 0(0%) | | 0.355# | |  | | |
| COPD | 2(20%) | | 0(0%) | 1(10%) | | 0.329# | |  | | |
| Preoperative Medication, no. (%) | 5(50%) | | 3(30%) | 1(10%) | | 0.149# | |  | | |
| ARB or ACEI | 1(10%) | | 3(30%) | 1(10%) | | 0.383# | |  | | |
| β-blockers | 0(0%) | | 0(0%) | 2(20%) | | 0.117# | |  | | |
| Calcium Channel Blockers | 0(0%) | | 1(10%) | 0(0%) | | 0.355# | |  | | |
| Statin use | 0(0%) | | 0(0%) | 1(10%) | | 0.355# | |  | | |
| Diuretics | 0(10%) | | 1(10%) | 0(0%) | | 0.355# | |  | | |
| Digoxin | 0(0%) | | 1(10%) | 0(0%) | | 0.355# | |  | | |
| Preoperative laboratory examination |  | |  |  | |  | |  | | |
| Hct [mean(SD); %] | 42.7 ± 5.7 | | 40.8 ± 3.2 | 39.6 ± 2.6 | | 0.245 | |  | | |
| PLt [mean(SD); 10^3^/mm^3^] | 173 ± 44 | | 187 ± 62 | 194 ± 38 | | 0.653 | |  | | |
| INR [mean(SD); s] | 1.06 ± 0.21 | | 1.08 ± 0.12 | 1.07 ± 0.17 | | 0.962 | |  | | |
| APTT [mean(SD); s] | 39 ± 5 | | 40 ± 5 | 40 ± 4 | | 0.884 | |  | | |
| TT [mean(SD); s] | 16.7 ± 1.3 | | 16.5 ± 1.0 | 16.4 ± 0.7 | | 0.798 | |  | | |
| PT [mean(SD); s] | 13.8 ± 2.0 | | 14.0 ± 1.2 | 13.9 ± 1.8 | | 0.936 | |  | | |
| PT % [mean(SD); %] | 97 ± 22 | | 90 ± 15 | 94 ± 21 | | 0.730 | |  | | |
| AST [mean(SD); U/L] | 29 ± 15 | | 25 ± 5 | 24 ± 9 | | 0.485 | |  | | |
| Blood glucose[mean(SD); mmol/L] | 4.8 ± 0.7 | | 4.8 ± 0.3 | 5.1 ± 0.9 | | 0.606 | |  | | |
| Creatinine [mean(SD); umol/L] | 57 ± 9 | | 58 ± 15 | 59 ± 14 | | 0.896 | |  | | |
| #: Fisher’s exact test was used; BMI= body mass index; ASA= American Society of Anesthesiologists; NYHA= New York Heart Association; EuroSCORE= European system for cardiac operative risk evaluation; AF= atrial fibrillation; COPD= chronic obstructive pulmonary disease; ARB= angiotensin receptor blockers; ACEI= angiotensin converting enzyme inhibitors; Hct= Hematocrit; PLt= platelet count; INR= international normalized ratio; APTT= activated partial thromboplastin time; TT= thrombin time; PT= Prothrombin time; AST= Aspartate aminotransferase. | | | | | | | | | | |

| Continue Supplemental Table 2. Demographic and surgical data. | | | | |  |
| --- | --- | --- | --- | --- | --- |
|  |  | placebo group  (n= 10) | low dose  group (n= 10) | high dose  group (n= 10) | *P*-value |
| Bleeding risk factors, ≤1/2-3/≥4, (n) |  | 9/1/0 | 10/0/0 | 9/1/0 | 0.585# |
| Type of surgery, no. (%) |  |  |  |  | 0.487# |
| Aortic valve |  | 4(40%) | 4(40%) | 3(30%) |  |
| Mitral valve |  | 2(20%) | 4(40%) | 1(10%) |  |
| Tricuspid valve |  | 0(0%) | 0(0%) | 1(10%) |  |
| Multiple valves |  | 4(40%) | 2(20%) | 5(50%) |  |
| Duration of anesthesia [mean(SD); min] |  | 267 ± 51 | 275 ± 24 | 288 ± 68 | 0.751 |
| Duration of surgery [mean(SD); min] |  | 226 ± 55 | 236 ± 27 | 247 ± 67 | 0.667 |
| CPB time [mean(SD); min] |  | 125 ± 44 | 117 ± 35 | 128 ± 41 | 0.828 |
| Heparin [mean(SD); mg] |  | 193 ± 36 | 217 ± 48 | 203 ± 46 | 0.491 |
| Protamine [mean(SD); mg] |  | 314 ± 45 | 357 ± 91 | 306 ± 55 | 0.205 |
| Input and Output Characteristics, [mean(SD); mL] |  |  |  |  |  |
| Input volume (Crystals and Colloid) |  | 1550 ± 497 | 1650 ± 474 | 1750 ± 354 | 0.611 |
| Cell salvage transfusion |  | 630 ± 206 | 605 ± 121 | 555 ± 222 | 0.666 |
| Blood loss |  | 255 ± 96 | 320 ± 63 | 330 ± 95 | 0.125 |
| Urine output |  | 1550 ± 497 | 1650 ± 474 | 1750 ± 354 | 0.452 |
| Intra-operative transfusion rate, no. (%) |  |  |  |  |  |
| Cell salvage |  | 3(10%) | 1(10%) | 3(30%) | 0.475 |
| RBCs transfusion |  | 2(20%) | 1(10%) | 2(20%) | 0.787 |
| FFP transfusion |  | 1(10%) | 1(10%) | 2(20%) | 0.749 |
| Platelet transfusion |  | 0(0%) | 0(0%) | 0(0%) | 1 |
| Fibrinogen, no. (%) |  | 1(10%) | 0(0%) | 0(0%) | 0.355 |
| #: Fisher’s exact test was used; CPB= cardiopulmonary bypass; RBCs= red blood cells; FFP= hematocrit; PLt= platelet count.  Prespecified risk factors for bleeding were an age of older than 70 years, female sex, use of low-molecular-weight heparin or an antiplatelet drug less than 5 days before surgery, renal impairment (estimated glomerular filtration rate, <60 ml per minute), and insulin-dependent diabetes. | | | | | |
