# Supplementary material for: Comparison of the in-vivo effect of two tranexamic acid doses on fibrinolysis parameters in adults undergoing valvular cardiac surgery with cardiopulmonary bypass - a pilot investigation
Source: BMC Anesthesiol. 2021 Feb 2;21:33. doi: 10.1186/s12871-021-01234-8 (PMC7852217; doi:10.1186/s12871-021-01234-8)
Supplement: Supplementary file 3 — Additional file 3: TableS3. Coagulation proteins without correcting for hemodilution at different time points between the three groups. [file 12871_2021_1234_MOESM3_ESM.doc]

| Supplemental Table 3: Coagulation proteins without correcting for hemodilution at different time points between the three groups. | | | | |  |
| --- | --- | --- | --- | --- | --- |
|  |  | placebo group  (n= 10) | low dose  group (n= 10) | high dose  group (n= 10) | *P*-value |
| PAI-1 [mean(SD); ng/mL] |  |  |  |  | 0.633 |
| T_1_ |  | 23.7 ± 2.5 | 22.9 ± 2.5 | 24.1 ± 2.2 |  |
| T_2_ |  | 27.0 ± 2.8 | 26.5 ± 2.7 | 26.4 ± 1.8 |  |
| T_3_ |  | 29.2 ± 2.1 | 28.7 ± 2.4 | 28.9 ± 3.4 |  |
| T_4_ |  | 30.9 ± 2.3 | 29.7 ± 2.5 | 28.9 ± 2.1 |  |
| T_5_ |  | 26.6 ± 1.8 | 27.3 ± 2.3 | 26.0 ± 1.6 |  |
| TAFI [mean(SD); ng/mL] |  |  |  |  | 0.070 |
| T_1_ |  | 17.5 ± 1.6 | 18.1 ± 1.1 | 16.4 ± 0.7 |  |
| T_2_ |  | 20.0 ± 0.9 | 20.9 ± 1.8 | 20.0± 1.3 |  |
| T_3_ |  | 22.3 ± 1.6 | 21.3 ± 1.7 | 20.4 ± 1.9 |  |
| T_4_ |  | 22.1 ± 1.8 | 21.0 ± 1.9 | 20.8 ± 2.2 |  |
| T_5_ |  | 20.9 ± 2.2 | 20.4 ± 2.0 | 19.6 ± 1.3 |  |
| PAP [mean(SD); ng/mL] |  |  |  |  | 0.143 |
| T_1_ |  | 13.9 ± 1.9 | 15.8 ± 1.5 | 13.3 ± 2.1 |  |
| T_2_ |  | 18.8 ± 1.9 | 19.4 ± 2.2 | 18.3 ± 2.3 |  |
| T_3_ |  | 21.7 ± 2.1 | 22.4 ± 2.4 | 22.3 ± 2.4 |  |
| T_4_ |  | 22.0 ± 2.9 | 22.7 ± 2.1 | 22.4 ± 2.3 |  |
| T_5_ |  | 19.3 ± 2.7 | 19.4 ± 1.7 | 19.6 ± 2.3 |  |
| tPA [mean(SD); ng/mL] |  |  |  |  | 0.018 |
| T_1_ |  | 3.0 ± 0.3 | 3.5 ± 0.4 | 3.3 ± 0.6 |  |
| T_2_ |  | 3.5 ± 0.4 | 4.1 ± 0.5 | 3.9 ± 0.3 |  |
| T_3_ |  | 4.1 ± 0.7 | 4.3 ± 0.7 | 4.2 ± 0.6 |  |
| T_4_ |  | 3.9 ± 0.6 | 4.2 ± 0.5 | 4.1 ± 0.3 |  |
| T_5_ |  | 3.50 ± 0.76 | 3.86 ± 0.23 | 3.84 ± 0.60 |  |
| TM [mean(SD); ng/mL] |  |  |  |  | 0.457 |
| T_1_ |  | 1.33 ± 0.09 | 1.55 ± 0.15 | 1.57 ± 0.14 |  |
| T_2_ |  | 1.62 ± 0.14 | 1.58 ± 0.16 | 1.60 ± 0.16 |  |
| T_3_ |  | 1.86 ± 0.15 | 1.85 ± 0.19 | 1.85 ± 0.18 |  |
| T_4_ |  | 1.70 ± 0.16 | 1.72 ± 0.14 | 1.86 ± 0.22 |  |
| T_5_ |  | 1.67 ± 0.18 | 1.56 ± 0.24 | 1.55 ± 0.14 |  |
| PAI-1: plasminogen activator inhibitor-1, TAFI: thrombin activatable fibrinolysis inhibitor, PAP: plasmin-antiplasmin complex, tPA: tissue plasminogen activator, TM: thrombomodulin. T_1_: per-operatively before TXA injection (baseline); T_2_: 5 min after TXA bolus administration (bolus); T_3_: 5 min after the onset of CPB (CPB); T_4_: 5 min before the end of CPB (End of CPB); T_5_: 5 min after protamine injection (protamine). | | | | | |
