# Supplementary material for: Comparison of the in-vivo effect of two tranexamic acid doses on fibrinolysis parameters in adults undergoing valvular cardiac surgery with cardiopulmonary bypass - a pilot investigation
Source: BMC Anesthesiol. 2021 Feb 2;21:33. doi: 10.1186/s12871-021-01234-8 (PMC7852217; doi:10.1186/s12871-021-01234-8)
Supplement: Supplementary file 4 — Additional file 4: TableS4. Platelet counts, standard coagulation test and TEG test at different time points between the three groups. [file 12871_2021_1234_MOESM4_ESM.doc]

| Supplemental Table 4: Platelet counts and standard coagulation test at different time points between the three groups. | | | | |  |
| --- | --- | --- | --- | --- | --- |
|  |  | placebo group  (n= 10) | low dose  group (n= 10) | high dose  group (n= 10) | *P*-value |
| PLt [mean(SD); 10^3^/mm^3^] |  |  |  |  | 0.699 |
| T_1_ |  | 195 ± 40 | 206 ± 70 | 218 ± 67 |  |
| T_2_ |  | 186 ± 44 | 199 ± 67 | 206 ± 65 |  |
| T_3_ |  | 97 ± 17 | 114 ± 44 | 107 ± 27 |  |
| T_4_ |  | 118 ± 30 | 137 ± 71 | 120 ± 39 |  |
| T_5_ |  | 99 ± 26 | 127 ± 60 | 106 ± 41 |  |
| INR [mean(SD); s] |  |  |  |  | 0.514 |
| T_1_ |  | 1.04 ± 0.05 | 1.07 ± 0.07 | 1.01 ± 0.05 |  |
| T_2_ |  | 1.07 ± 0.07 | 1.09 ± 0.06 | 1.05 ± 0.05 |  |
| T_3_ |  | 2.32 ± 0.56 | 2.09 ± 0.25 | 2.11 ± 0.23 |  |
| T_4_ |  | 2.04 ± 0.23 | 1.95 ± 0.17 | 2.03 ± 0.22 |  |
| T_5_ |  | 1.78 ± 0.19 | 1.73 ± 0.19 | 1.73 ± 0.19 |  |
| PT [mean(SD); s] |  |  |  |  | 0.632 |
| T_1_ |  | 13.7 ± 0.6 | 13.9 ± 0.7 | 13.3 ± 0.5 |  |
| T_2_ |  | 13.9 ± 0.7 | 14.1 ± 0.6 | 13.7 ± 0.5 |  |
| T_3_ |  | 25.2 ± 5.0 | 23.3 ± 2.4 | 23.7 ± 2.1 |  |
| T_4_ |  | 22.8 ± 2.0 | 22.1 ± 1.5 | 23.0 ± 2.1 |  |
| T_5_ |  | 20.5 ± 1.6 | 20.1 ± 1.7 | 20.3 ± 1.7 |  |
| PT % [mean(SD); %] |  |  |  |  | 0.561 |
| T_1_ |  | 93 ± 8 | 91 ± 9 | 99 ± 8 |  |
| T_2_ |  | 90 ± 9 | 87 ± 8 | 92 ± 7 |  |
| T_3_ |  | 34 ± 8 | 37 ± 5 | 36 ± 5 |  |
| T_4_ |  | 38 ± 5 | 40 ± 4 | 38 ± 5 |  |
| T_5_ |  | 45 ± 6 | 47 ± 6 | 46 ± 6 |  |
| APTT [mean(SD); s] |  | 38.6 ± 4.5 | 38.3 ± 2.9 | 37.8 ± 2.8 | 0.790 |
| T_1_ |  | 37.8 ± 4.0 | 38.5 ± 3.1 | 38.2 ± 2.6 |  |
| T_2_ |  | 48 ± 8 | 45 ± 5 | 49 ± 6 |  |
| T_5_ |  |  |  |  | 0.603 |
| TT [mean(SD); s] |  | 17.4 ± 2.5 | 17.0 ± 1.3 | 16.7 ± 0.7 |  |
| T_1_ |  | 16.4 ± 0.9 | 16.7 ± 0.7 | 16.7 ± 0.5 |  |
| T_2_ |  | 18.5 ± 1.6 | 19.0 ± 1.8 | 17.9 ± 1.1 |  |
| T_5_ |  |  |  |  |  |
| PLt= platelet count; PT= Prothrombin time; APTT= activated partial thromboplastin time; TT= thrombin time; T_1_= per-operatively before TXA injection (baseline); T_2_= 5 min after TXA bolus administration (bolus); T_3_= 5 min after the onset of CPB (CPB); T_4_= 5 min before the end of CPB (End of CPB); T_5_= 5 min after protamine injection (protamine). | | | | | |
