# Supplementary material for: Comparison of the in-vivo effect of two tranexamic acid doses on fibrinolysis parameters in adults undergoing valvular cardiac surgery with cardiopulmonary bypass - a pilot investigation
Source: BMC Anesthesiol. 2021 Feb 2;21:33. doi: 10.1186/s12871-021-01234-8 (PMC7852217; doi:10.1186/s12871-021-01234-8)
Supplement: Supplementary file 5 — Additional file 5: TableS5. Postoperative clinical data and Standard coagulation test. [file 12871_2021_1234_MOESM5_ESM.doc]

| Supplemental Table 5: Postoperative clinical data and Standard coagulation test | | | | | | | | | |  | |
| --- | --- | --- | --- | --- | --- | --- | --- | --- | --- | --- | --- |
|  | |  | | placebo group  (n= 10) | | low dose  group (n= 10) | | high dose  group (n= 10) | | *P*-value | |
| **Input in the first post-operative morning** [mean(SD); mL] | |  | | 2054 ± 549 | | 2105 ± 654 | | 2217 ± 762 | | 0.853 | |
| **postoperative transfusion**, no. (%) | | | | | | | | | |  | |
| RBCs transfusion, no. (%) | |  | |  | |  | |  | |  | |
| FFP transfusion, no. (%) | |  | | 0(0%) | | 0(0%) | | 1(10%) | | 0.355 | |
| Platelet transfusion, no. (%) | |  | | 0(0%) | | 0(0%) | | 1(10%) | | 0.355 | |
| **Postoperative coagulation drugs** | | | | | |  | |  | |  | |
| PCC, no. (%) | |  | | 0(0%) | | 0(0%) | | 1(10%) | | 0.355 | |
| Fibrinogen, no. (%) | |  | | 0(0%) | | 0(0%) | | 1(10%) | | 0.355 | |
| TXA, no. (%) | |  | | 1(10%) | | 1(10%) | | 2(20%) | | 0.749 | |
| **Laboratory Characteristics in the first post-operative morning** | | | | | | | | | |  | |
| Hct [mean(SD); %] | | |  | | 31 ± 4 | | 32 ± 4 | | 29 ± 5 | | 0.233 |
| PLt [mean(SD); 10^3^/mm^3^] | | |  | | 103 ± 32 | | 125 ± 48 | | 90 ± 29 | | 0.123 |
| PT [mean(SD); s] | | |  | | 15.4 ± 1.4 | | 15.1 ± 1.2 | | 15.5 ± 2.5 | | 0.881 |
| PT % [mean(SD); %] | | |  | | 75 ± 13 | | 76 ± 12 | | 76 ± 18 | | 0.965 |
| INR [mean(SD); s] | | |  | | 1.22 ± 0.13 | | 1.19 ± 0.12 | | 1.23 ± 0.26 | | 0.881 |
| APTT [mean(SD); s] | | |  | | 48 ± 6 | | 46 ± 12 | | 46 ± 14 | | 0.960 |
| TT [mean(SD); s] | | |  | | 20 ± 7 | | 22 ± 12 | | 19 ± 7 | | 0.822 |
|  | RBCs= red blood cells; FFP= hematocrit; PCC= Prothrombin Complex Concentrate; TXA= Tranexamic acid; Hct= Hematocrit; PLt= platelet count; INR= international normalized ratio; APTT= activated partial thromboplastin time; TT= thrombin time; PT= Prothrombin time. | | | | | | | | | | |
